# Adherence to the Mediterranean Diet in Preventing Major Cardiovascular Events in Patients with Ischemic Heart Disease: The EVA Study

**DOI:** 10.3390/nu15143150

**Published:** 2023-07-14

**Authors:** Roberto Cangemi, Marzia Miglionico, Tania D’Amico, Salvatore Fasano, Marco Proietti, Giulio Francesco Romiti, Bernadette Corica, Lucia Stefanini, Gaetano Tanzilli, Stefania Basili, Valeria Raparelli, Maria Grazia Tarsitano, EVA Collaborative Group

**Affiliations:** 1 Department of Translational and Precision Medicine, Sapienza University of Rome, 00185 Rome, Italy; 2 Department of Clinical Sciences and Community Health, University of Milan, 20122 Milan, Italy; 3 Division of Subacute Care, IRCCS Istituti Clinici Scientifici Maugeri, 20138 Milan, Italy; 4 Department of Internal Medicine, Anesthesiologic and Cardiovascular Sciences, University Sapienza, 00161 Rome, Italy; 5 Department of Experimental Medicine, Sapienza University of Rome, 00161 Rome, Italy; 6 Department of Translational Medicine, University of Ferrara, 44121 Ferrara, Italy; 7 University Center for Studies on Gender Medicine, University of Ferrara, 44121 Ferrara, Italy; 8 Faculty of Nursing, University of Alberta, Edmonton, AB T6G 2R3, Canada; 9 Department of Medical and Surgical Sciences, Magna Graecia University, 88100 Catanzaro, Italy

**Keywords:** Mediterranean diet, ischemic heart disease, adherence, sex, gender

## Abstract

Background: Adherence to healthy dietary patterns, such as the Mediterranean diet (Med-diet), is recommended for the maintenance of cardiovascular health. The determinants for adherence to Med-diet and its importance in secondary cardiovascular disease prevention are still unclear. The aim of the study was to evaluate the influence of sex- and psycho-socio-cultural (i.e., gender-related) factors on Med-diet adherence and its role in preventing major cardiovascular events (MACEs) in patients with ischemic heart disease (IHD). Methods: Med-diet adherence was evaluated among 503 consecutive adults with IHD. MACEs were collected during a long-term follow-up. Results: Male Bem Sex-Role Inventory score (i.e., male personality traits) and physical functional capacity were associated with higher adherence, while cohabitation with a smoker and physical inactivity with poorer adherence. During a median follow-up of 22 months, 48 participants experienced MACEs (17.5%, 8.1%, and 3.9% of patients with low, medium, and high adherence, respectively; *p* = 0.016). At multivariate Cox--regression analysis, a greater adherence remained inversely associated with MACEs (HR: 0.49; 95% CI: 0.29–0.82; *p* = 0.006) after adjusting for confounding factors. Conclusion: The study suggests that gender-related factors have a role in maintaining a healthy dietary pattern. Improving Med-diet adherence may lower the risk of recurring cardiovascular events.

## 1. Introduction

The development and progression of cardiovascular disease (CVD) are closely associated with poor diet habits [1]. Thus, current American and European guidelines strongly recommend following dietary patterns that are most beneficial in reducing cardiometabolic risk, i.e., fruits and vegetables; whole grains food; mostly protein from plants, fish, and seafood; low-fat dairy products; and limited consumption of alcoholic beverages [1,2].

Among the known dietary patterns, the Mediterranean diet (Med-diet) is the one that comes closest to the characteristics listed above. In the past decades, the Med-diet has been recognized as a gold standard in healthy eating since a greater adherence to the Med-diet has been associated with a reduction in morbidity and mortality, especially from cardiovascular and cerebrovascular causes [3].

Despite the protective role of the Med-diet against CVD, a surprising shift away from the traditional Med-diet has occurred mainly in most Mediterranean countries [4,5,6]. The reduction in adherence could be influenced by numerous factors. Most clinical studies have studied predominantly the biological determinants of adherence, including sex, obesity, and diabetes [6,7,8,9]. However, psychosocial factors seem to be of crucial importance, for example, low economic status [10]. Although biological sex (i.e., sex assigned at birth) and socio-cultural gender (i.e., psycho-socio-cultural attributes of a person) may play an important role in adherence, both aspects are generally overlooked and underreported [11,12,13].

Throughout the last decade, it has become clearer to both practicing clinicians and researchers that the integration of gendered factors (i.e., gender identity, gender role, gender relations, and institutionalized gender) is pivotal to obtaining high-quality, equitable, and inclusive evidence. In fact, the gendered factors have a different distribution between men and women, and they intersect with the biological features linked to biological sex in shaping health and diseases [12]. We previously reported how the association of sex- and gender-related factors varies according to the degree of adherence to the Med-diet in a preliminary analysis on a cohort of adults undergoing coronary angiography for ischemic heart disease (IHD). Specifically, proxies of gender identity (i.e., fewer male personality traits and high perceived stress) were associated with low Med-diet adherence regardless of sex, age, and comorbidities [14].

Furthermore, while the evidence of a protective effect of a Med-diet pattern in the primary prevention of CVD is consistent, evidence of such an effect in secondary prevention (i.e., in patients with known IHD) still deserves attention, especially in the contemporary era and considering the advancement of percutaneous and pharmacological treatments [15]. In this regard, two recent studies revealed sex-related disparities in the role of nutrition. Specifically, one study found that, in patients with acute coronary syndrome, malnutrition was a predictor of in-hospital mortality only in women [16], while the other study found that, in patients with heart failure (HF), malnutrition was independently associated with the odds of in-hospital mortality in men, but not in women [17].

Thus, we expanded our prior work by providing data on the complete cohort of patients enrolled in the EVA study, with the aim to evaluate the influence of sex- and psycho-socio-cultural (i.e., gender-related) factors on Med-diet adherence and its role in preventing major cardiovascular events (MACEs) in patients with ischemic heart disease (IHD).

## 2. Materials and Methods

### 2.1. Study Population

Data for the present analysis were obtained from the “Endocrine Vascular disease Approach” (EVA) project (ClinicalTrials.gov identifier NCT02737982), which is an observational registry of female and male individuals (>18 years) referred to the cardiac catheterization laboratory to undergo coronary angiography and/or percutaneous coronary intervention for suspected ischemic heart disease (IHD). The study includes individuals with obstructive and non-obstructive coronary artery disease (CAD). The main exclusion criteria were: life expectancy less than 12 months, active cancer (i.e., chemotherapy or ≤5 years from diagnosis), pregnancy, previous coronary artery bypass graft, documented moderate-severe valvular heart disease, and biological or mechanical valves. The EVA study design was previously published [18]. 

The current analysis included all participants recruited between April 2015 and January 2020. The participants’ characteristics were obtained through a combination of medical record review and standardized in-person interviews conducted by trained personnel. Baseline clinical characteristics that were collected included age, as well as the presence of traditional risk factors, like smoking, hypertension, and dyslipidemia. Height, body weight, body mass index (BMI), and blood pressure were measured at hospital admission. Additionally, concomitant diseases such as type 2 diabetes (T2DM), prior myocardial infarction (MI), HF, and cerebral vasculopathy (prior stroke or transient ischemic attack (TIA)) were collected. Functional status was assessed using the Duke Activity Status Index (DASI), a brief, self-administered questionnaire that measures functional capacity and assesses aspects of the quality of life of patients with CVD [19]. The type of CAD was determined based on angiography, with coronary obstruction <50% indicating non-obstructive CAD and coronary obstruction ≥50% obstructive CAD).

In accordance with the gender domain definition proposed by the Women’s Health Research Network [20], the study considered the following gender-related factors for each participant: (i) gender relations: marital status (i.e., married/living with partner vs. others) and social and emotional support; (ii) gender roles: primary earner status and employment within the household; (iii) institutionalized gender: less than secondary school education level and low household income (<1000 Euros per month); and (iv) gender identity: Bem Sex Role Inventory (BSRI) [21]. Finally, the level of stress was evaluated by the Perceived Stress Scale-10 items (PSS-10) [22]. The BSRI and the PSS-10 are self-reported questionnaires. The BSRI is based on gender stereotypes (masculine, feminine, or neutral) and measures how well a person fits into traditional sex roles [21]. The PSS-10 explores the individuals’ perception of their experienced stress in the last month [22].

Moreover, risk-taking behaviors such as physical inactivity (defined as no recreational activity or less than once per week) and cohabitation with a smoker were also explored. 

The study was conducted in accordance with the Declaration of Helsinki and was approved by the local Ethics Committee. Written informed consent was obtained from all participants. 

### 2.2. Assessment of Adherence to Med-Diet

The level of adherence to the Med-diet, which relied on self-motivation, was evaluated upon admission using a self-administered questionnaire that had undergone prior validation [23]. The questionnaire consisted of 14 items. Patients were aided by a trained dietitian at the time of enrollment to ensure accurate completion of the questionnaire. Points were assigned based on how often a specific food was eaten per day/week, according to the Med-diet principles. The questions were aimed at obtaining daily consumption of the following food/nutrients: olive oil; vegetables; fruit or natural fruit juices; red meat; butter; margarine or cream; sweetened and/or carbonated beverages; wine; legumes; fish or shellfish; commercial sweets or pastries (not homemade); nuts (including peanuts); chicken, turkey or rabbit; red meat; and sofrito (tomato sauce with onions, leeks or garlic and simmered in olive oil). 

A score of equal to or less than 5, as obtained from the sum of the 14 responses, defined a low adherence; a score between 6 and 9 defined medium adherence; a score equal or superior to 10 defined high adherence.

### 2.3. Follow-Up

Participants were followed up by phone and outpatient visits, when available, at least one year after discharge. After one year, phone interviews were conducted periodically every three months. In the case of clinical adverse events, their adjudication was performed through medical chart reviews. The main outcomes assessed were the occurrence of any major adverse cardiovascular event (MACE), including cardiovascular death, MI, stroke/TIA.

### 2.4. Statistical Analysis

Categorical variables were reported as counts and percentages, while continuous variables as mean ± standard deviation (SD) or medians and interquartile ranges (IQRs). The chi-square or Fisher’s exact test was used to evaluate differences between percentages. All continuous variables were tested for normality with the Shapiro–Wilk test. For normally distributed continuous variables, the unpaired Student’s *t*-test and ANOVA were employed, while appropriate nonparametric tests, such as the Mann–Whitney and Kruskal–Wallis tests, were used for all other variables. The bivariate and multivariate effects of prognostic factors on high adherence to Med-diet were assessed by means of logistic regression models. Wald confidence intervals and tests for odds ratios and adjusted odds ratios were computed based on the estimated standard errors. The stochastic level of entry into the multivariable model was set at 0.10. To estimate the cumulative incidence after grouping the population, the Kaplan–Meier product–limit estimator was utilized. The log-rank test was employed to formally compare survival curves. In order to determine the adjusted relative risks of outcome events for each clinical variable, Cox proportional hazards analysis was conducted. For multivariate models, model selection was performed using forward stepwise regression based on the Akaike information criterion.

Only *p* values < 0.05 were considered statistically significant. All tests were 2-tailed, and analyses were performed using computer software packages (IBM SPSS Statistics, ver. 27).

## 3. Results

### 3.1. Baseline Participants’ Characteristics and Med-Diet Adherence

**Figure 1:**
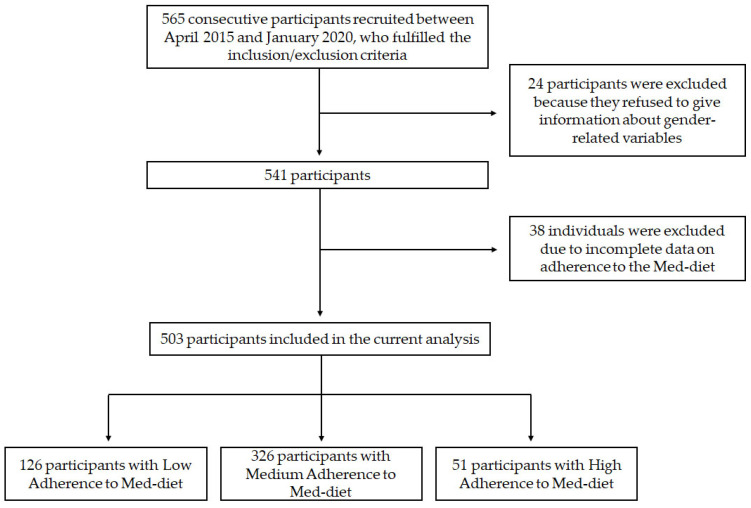
Flowchart of the study.

Among 565 consecutive participants recruited between April 2015 and January 2020, a total of 503 (29% female sex, age: 66.8 ± 11.2 years) participants were included in the present analysis (Figure 1). Within this cohort, low adherence was reported in 126 (25%) cases, medium adherence in 326 (65%), and high adherence in 51 (10% cases).

The baseline clinical characteristics of the included cohort of patients are reported in Table 1.

No statistically significant differences were observed between subjects with low, medium, and high adherence with respect to age, sex, BMI, smoking habit, major cardiovascular comorbidities, and hypertension, even if the female sex showed a tendency to be less prevalent in participants with high adherence to Med-diet (*p* = 0.053, compared to participants with low and medium adherence). Physical inactivity was progressively less common in patients with medium and high adherence, while the DASI score was higher in patients with high adherence (Table 1).

Coronary angiographic characteristics of EVA patients, according to the type of adherence to the Med-diet, are reported in Table 2. No statistically significant differences were observed regarding the type of CAD (i.e., non-obstructive vs. obstructive) and the type of clinical presentation (i.e., acute vs. chronic), even if a non-significant progressive decrease in obstructive CAD and acute clinical presentation was found with the increase adherence to the Med-diet.

The distribution of gender-related factors according to Med-diet adherence is shown in Table 3.

Overall, marital status, employment, less than secondary school education level attainment, and poor social support did not differ among the groups. Similarly, no differences were found in the Perceived Stress Scale 10 (PSS-10) score. Conversely, male BSRI progressively increased according to a higher Med-diet adherence, and “living with a smoker” was progressively less common in patients with medium and high adherence.

In order to further characterize the relationship between clinical and gender-related variables and adherence to the Med-diet, we conducted a logistic regression analysis to evaluate predictors of higher adherence, including sex, physical inactivity, DASI score, male BSRI, and living with a smoker.

The multivariable model revealed that only male BSRI (odd ratio (OR): 1.506; 95%; confidence interval (CI): 1.022–2.218; *p* = 0.038) remained significantly associated with high adherence (as opposed to medium and low adherence), independently from male sex (OR: 2.273; 95% CI: 0.916–5.636; *p* = 0.076), physical inactivity (OR: 0.743; 95% CI: 0.355–1.553; *p* = 0.429), DASI score (OR: 1.470; 95% CI: 0.992–1.033; *p* = 0.255), and living with a smoker (OR: 0.702; 95% CI: 0.337–1.463; *p* = 0.345).

### 3.2. Adherence to Med-Diet and MACEs at Follow-Up

Four-hundred seventy-nine participants were followed for a median time of 22 months (interquartile range: 11–37 months), yielding a total of 978 person-years of observation.

Twenty-four participants (4.7% of the whole cohort) were lost during the follow-up.

**Figure 2:**
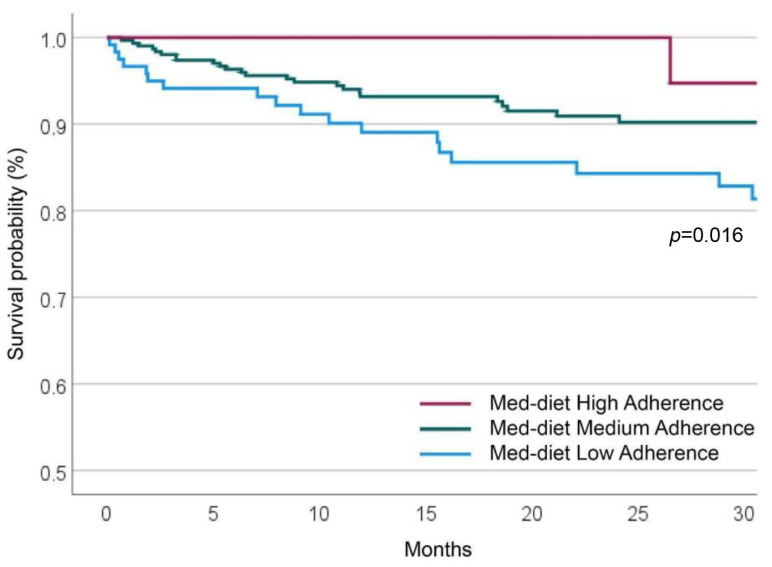
Cumulative MACE survival probability at long-term follow-up, according to Med-diet Adherence.

During the follow-up, 48 participants experienced a MACE (16 non-fatal MI, 10 non-fatal strokes, and 22 cardiovascular deaths). MACEs during the follow-up were more common in participants with low adherence to the Med-diet (*n* = 21 out of 120 participants, 17.5%) and progressively less common in patients with medium (*n* = 25 out of 308 participants, 8.1%) and high adherence (*n* = 2 out of 51 participants, 3.9%) (Figure 2).

At Cox-regression univariate analyses, age, HF, T2DM, and obstructive CAD were directly associated with MACEs, while DASI score and Med-diet adherence were inversely associated (Table 4).

At a multivariate COX-regression analysis, HF, obstructive CAD, and T2DM were directly associated with MACE, while the increasing Med-diet adherence was inversely associated with MACE (Table 5).

## 4. Discussion

The present study shows that, in individuals with ischemic heart disease (IHD), Med-diet adherence is influenced by gender-related factors and has an important impact on major adverse cardiac events (MACEs) during a 2-year follow-up.

The Med-diet has been associated with numerous health benefits, including a lower risk of CVD, cancer, and other chronic diseases. For those reasons, the ESC guidelines encourage to adopt a Med-diet to lower CVD risk [2]. Despite the current indications, in the last decades, adherence to the Med-diet decreased, especially in Mediterranean countries [4,5,6]. The mechanisms underlining this decline have not been well clarified: adherence to Med-diet varies a lot between individuals and may be influenced by a range of factors, including gender-related ones. In this context, the findings of our study highlight the importance of promoting adherence to the Med-diet in individuals with IHD as a means of reducing the risk of MACEs. Furthermore, the study suggests that gender-related factors may need to be considered when designing interventions aimed at promoting adherence to the Med-diet.

The Med-diet is considered an outstanding common health pattern in dietary epidemiology and has been extensively studied. It is defined as a traditional dietary pattern that was prevalent among people living in the Mediterranean region in the 1950s and 1960s. At that time, the main feature of this diet was low consumption of meat and meat products, with very low consumption of red meat and little or no consumption of processed meat, butter, sweets, or other whole dairy products. In contrast, there was high consumption of olive oil, locally grown minimally processed vegetables, fruits, nuts, legumes, and grains (mostly unrefined). A great source of protein was fish, depending on their proximity to the sea. The generous use of olive oil in traditionally cooked vegetables, legumes, and salads, combined with moderate consumption of red wine with meals, made this diet both nutritious and palatable [24]. Several studies have shown that adherence to the Med-diet could lead to a reduction in morbidity and mortality from CVD.

A systematic review ranked the Med-diet as the dietary pattern most likely to prevent CAD [3]. A meta-analysis involving over 1 million individuals supported the relationship between adherence to the Med-diet and a reduction in all-cause mortality and mortality from CVD [25]. In the Spanish landmark PREDIMED trial, involving 7447 persons at high cardiovascular risk, a 5-year intervention with a Med-diet significantly reduced the incidence of major cardiovascular events, which included non-fatal stroke, non-fatal CAD, and all fatal CVD events [26].

Despite the fact that the beneficial effects of Med-diet in primary prevention are generally recognized, its role in secondary prevention, i.e., in individuals who have already experienced a CVD, is still a matter of debate. In the late 1990s, the Lyon Diet Heart study demonstrated the potential of Med-diet in reducing the risk of cardiovascular events in both primary and secondary prevention [27], but most recent evidence suggests that the benefits of lifestyle and diet might be less significant in patients who have undergone coronary angiography with or without percutaneous coronary intervention, due to the use of modern treatments for CAD [28], such as new antithrombotic agents and newer and more potent lipid-lowering drugs.

In this perspective, a recent Cochrane systematic review showed limited evidence regarding the effects of a Med-diet on clinical endpoints and CVD risk factors for secondary prevention [15]. Despite this, the CORDIOPREV trial reported that Med-diet was superior to a low-fat diet in preventing major cardiovascular events in secondary prevention of CVD, even though this effect was more evident in males compared to females [29].

In our cohort of patients undergoing coronary angiography, we found that higher adherence to Med-diet was associated with a lower risk of MACE during a long-term follow-up, independently from age, sex, known cardiovascular risk factors, DASI, presentation (acute vs. non-acute coronary syndrome) and type of CAD (obstructive vs. non-obstructive).

Thus, our study suggests that adherence to Med-diet remains a cornerstone in preventing cardiovascular events after coronary angiography, even in the context of modern treatments for IHD.

The peculiarity of our study was that we evaluated the potential impact of different types of IHD (obstructive vs. non-obstructive; acute vs. non-acute IHD) together with sex and gender-related variables.

In this regard, we found that people who reported low adherence to the Med-diet were more likely to be physically inactive and more likely to live with a smoker. On the other hand, those with higher adherence to the Med-diet were more likely to exhibit masculine personality traits, such as assertive and competitive attitude, risk-taking, independence, and individualism (all items of the male BSRI score). The study also suggested that physical functional capacity tended to accompany a healthier diet, as those with higher DASI scores had a higher adherence to the Med-diet.

Physical functioning is a multifaceted factor that can be impacted by a range of co-occurring conditions, such as the patient’s baseline functional level, the severity of their heart disease, other concurrent medical conditions, and social or psychological barriers. Additionally, psychosocial factors, such as depression, anxiety, and the level of social support available, can also have an impact.

Thus, our study suggests that adherence to a healthy diet is the result of a complex interaction between various factors, including personal inclinations, coping strategies for stress, physical activity, and the ability to lead a full and independent life.

This analysis confirms and extends our previous report showing that gender-related factors could influence adherence to the Med-diet [14].

Differently from our previous report [14], active smoking was not significantly associated with a low Med-diet adherence, even if a tendency to a lower adherence was found in smokers. This difference could depend on the cohort that was larger than the previous one. However, in this new analysis, we found that a gender-sensitive variable (i.e., “living with a smoker”) was associated with a lower adherence to the Med-diet, highlighting the role of psychological and socio-cultural factors in healthy behaviors.

In this study, we performed a multivariable logistic regression analysis, specifically aimed to identify predictors of high adherence to Med-diet (as opposed to low and medium adherence), that showed that a higher male BSRI was associated with higher adherence, independently to the biological sex. Thus, a higher male BSRI identified both men and women who were more likely to exhibit masculine traits and to show a higher adherence to the Med-diet. This finding underlines the opportunities that derive from disentangling sex and gender when it comes to healthy lifestyle behaviors. Beyond clinical features of individuals, multiple non-biological factors, including personality traits, can influence adherence to the Med-diet. A paradigm shift in the design of gendered and tailored interventions for improving health cardiovascular outcomes is very much needed to pursue the precision delivery of health solutions. In the field of cardiovascular prevention, addressing these factors could potentially improve adherence and overall health outcomes.

Some important limitations of this study should be noted. One of the limitations is that as it was an observational study, there may be other factors that were not measured and could have influenced the results. Additionally, the sample size was relatively small, and the EVA study was not specifically designed to assess the impact of gender on adherence to the Med-diet or its effect on MACEs [18,30]. Furthermore, we did not collect data on total energy intake from food; thus, we could not adjust our results accordingly, even if energy intake is considered an essential factor in CVD prevention [31]. Moreover, the study participants were a selected group of individuals at high cardiovascular risk hospitalized for IHD from a single center in Italy; hence the results may not be generalizable to other populations. In this regard, the patients’ selection in the EVA study (patients undergoing coronary angiography and/or percutaneous coronary intervention for IHD) implies that this group of patients may have more risk factors than the general population. The incidence of cardiovascular risk factors such as dyslipidemia, T2DM, and smoking were actually particularly high, as was the incidence of previous ischemic heart disease or cerebrovascular disease. However, this kind of selection is of particular interest because the benefits of the Med-diet are not yet fully understood in this context.

Finally, the number of events during follow-up was relatively low. Therefore, new studies with a larger sample size and/or longer follow-ups are needed to confirm our data and to identify new strategies to improve adherence to a healthier lifestyle in these patients.

Despite these limitations, the study presents several notable strengths. Firstly, it highlights the potential impact of gender-related factors on the adoption of a healthy lifestyle, like Med-diet adherence. Consequently, healthcare professionals should consider patients’ personality traits to enhance and encourage healthy behaviors, particularly in individuals at high risk of CVD. Secondly, it provides valuable insights into the potential benefits of adhering to the Med-diet within the context of modern treatments for IHD.

## 5. Conclusions

In conclusion, our study suggests that higher adherence to the Med-diet is associated with a lower risk of cardiovascular events in patients undergoing coronary angiography, even in the context of modern treatments for ischemic heart disease. Gendered factors related to personality traits and lifestyle may have an important role in maintaining a healthy dietary pattern. Identifying patients at higher risk of low adherence based on non-traditional, gender-based aspects of patients should be promoted in clinical practice. Fostering strategies to improve Med-diet adherence is of pivotal importance in IHD patients as a higher Med-diet adherence was associated with a lower risk of MACE during a long-term follow-up. Our data highlight how nutritional counseling should be promoted as a secondary prevention intervention to improve the long-term outcome of IHD.

Gender, as a determinant of health status, has been largely overlooked in clinical research. Therefore, new studies are needed to evaluate the effects of individual gender-related factors on participants’ attitudes toward adherence to healthy dietary patterns. Such studies would help identify new non-traditional risk factors and ultimately reduce the risk of cardiovascular disease.

## Figures and Tables

**Table 1:** Baseline clinical characteristics of participants according to the adherence to the Med-diet.

Variables	Low Adherence (*n* = 126)	Medium Adherence (*n* = 326)	High Adherence (*n* = 51)	*p*
Age, years	65.7 ± 11.9	67.4 ± 11.1	66.3 ± 9.5	0.353
BMI, kg/m^2^	27.2 ± 4.2	26.9 ± 4.5	26.8 ± 4.0	0.824
Female sex, *n* (%)	37 (29%)	102 (31%)	9 (18%)	0.139
Previous MI, *n* (%)	31 (25%)	85 (26%)	8 (16%)	0.278
HF, *n* (%)	14 (11%)	48 (15%)	5 (10%)	0.442
Hypertension, *n* (%)	103 (82%)	259 (79%)	41 (80%)	0.859
Dyslipidemia, *n* (%)	66 (52%)	167 (51%)	24 (47%)	0.811
T2DM, *n* (%)	37 (29%)	86 (26%)	9 (18%)	0.275
Stroke/TIA, *n* (%)	13 (10.3%)	39 (12%)	7 (14%)	0.796
Active smoking, *n* (%)	36 (29%)	85 (26%)	9 (18%)	0.319
Physical inactivity, *n* (%)	108 (87%)	249 (77%)	35 (69%)	0.013
DASI (median and IQR)	38 (23–58)	33 (19–50)	45 (27–58)	0.046

BMI, body mass index; HF, heart failure; MI, myocardial infarction; DASI, Duke Activity Status Index; T2DM, type 2 diabetes; TIA, transient ischemic attack. Data are presented as number of patients and percentage, mean ± SD, or median (IQR).

**Table 2:** Angiographic characteristics of participants, according to the adherence to the Med-diet.

Variables	Low Adherence (*n* = 126)	Medium Adherence (*n* = 326)	High Adherence (*n* = 51)	*p*
Obstructive CAD	98 (78%)	234 (72%)	34 (67%)	0.254
Presentation as ACS	68 (54%)	160 (49%)	19 (38%)	0.161

ACS, acute coronary syndrome; CAD, coronary artery disease. Data are presented as number of patients and percentage or as mean ± SD or median [IQR].

**Table 3:** Gender-related factors according to the adherence to the Med-diet.

Variables	Low Adherence (*n* = 126)	Medium Adherence (*n* = 326)	High Adherence (*n* = 51)	*p*
Married/living with partner, *n* (%)	95 (75%)	222 (68%)	36 (71%)	0.314
Male BSRI (mean ± SD)	4.85 ± 1.13	4.94 ± 0.91	5.31 ± 0.74	0.031
Female BSRI (mean ± SD)	5.71 ± 0.72	5.84 ± 0.75	5.91 ± 0.72	0.349
Neutral BSRI (mean ± SD)	4.84 ± 0.67	4.84 ± 0.66	4.85 ± 0.57	0.993
PSS-10 (median and IQR)	19 (11–22)	16 (12–21)	15 (10–22)	0.362
Living with a smoker, *n* (%)	14 (11%)	22 (7%)	1 (2%)	0.001
Active employment, *n* (%)	50 (40%)	118 (36%)	23 (45%)	0.396
Less than secondary school education level, *n* (%) *	29 (33%)	88 (35%)	16 (35%)	0.886
Low social support, *n* (%) *	11 (13%)	21 (8%)	5 (11%)	0.392

BSRI, Bem Sex Role Inventory. “Male BSRI” encompasses masculine traits; “Female BSRI” encompasses feminine traits; “Neutral BRSI” encompasses filler traits thought to be gender neutral [21]. Data are presented as number of patients and percentage, mean ± SD, or median (IQR). * data available for 383 participants.

**Table 4:** Factors associated with MACEs during the follow-up. Univariate analyses.

Variables	HR	95% CI		*p*
Female sex	1.098	0.602	2.002	0.760
Age	1.044	1.014	1.075	0.004
BMI	0.962	0.898	1.031	0.273
Previous MI	1.148	0.607	2.170	0.671
HF	4.119	2.293	7.398	<0.001
Hypertension	2.146	0.850	5.418	0.106
Dyslipidemia	1.049	0.595	1.851	0.869
T2DM	2.249	1.271	3.979	0.005
Stroke/TIA	1.116	0.474	2.626	0.801
Active smoking	1.201	0.644	2.238	0.564
Physical inactivity	1.616	0.725	3.603	0.241
Living with a smoker	1.056	0.574	1.946	0.860
DASI	0.976	0.959	0.992	0.004
Male BSRI	1.421	0.903	2.236	0.129
Med-diet Adherence	0.492	0.300	0.809	0.005
Obstructive CAD	3.909	1.404	10.881	0.009
Presentation as ACS	1.394	0.780	2.491	0.262

ACS, acute coronary syndrome; BMI, body mass index; Male BSRI, Male Bem Sex Role Inventory (encompasses masculine traits) [21]; CAD, coronary artery disease; CI, confidence interval; DASI, Duke Activity Status Index; HF, heart failure; HR, hazard ratio; MI, myocardial infarction; TIA, transient ischemic attack; T2DM, type 2 diabetes mellitus. DASI. Male BSRI” encompasses masculine traits.

**Table 5:** Factors associated with MACEs during the follow-up. Multivariate COX-regression analysis.

Variables	HR	95% CI		*p*
Obstructive CAD (vs. non-obstructive CAD)	3.069	1.097	8.586	0.033
HF	3.695	2.029	6.728	<0.001
T2DM	1.887	1.058	3.364	0.031
Med-diet Adherence (high and medium vs. low adherence)	0.491	0.295	0.818	0.006

After adjusting for age, sex, BMI, DASI score. CAD, coronary artery disease; CI, confidence interval; HF, heart failure; HR, hazard ratio; T2DM, type 2 diabetes.

## Data Availability

The data presented in this study are available from the corresponding author upon reasonable request.
